# Supplementary material for: Exploring Plastomic Resources in Sempervivum (Crassulaceae): Implications for Phylogenetics
Source: Genes (Basel). 2024 Mar 30;15(4):441. doi: 10.3390/genes15040441 (PMC11049882; doi:10.3390/genes15040441)
Supplement: Supplementary file 1 [file genes-15-00441-s001.zip › (revised)supplementary files_Junhu Kan/Figure S1.pdf]

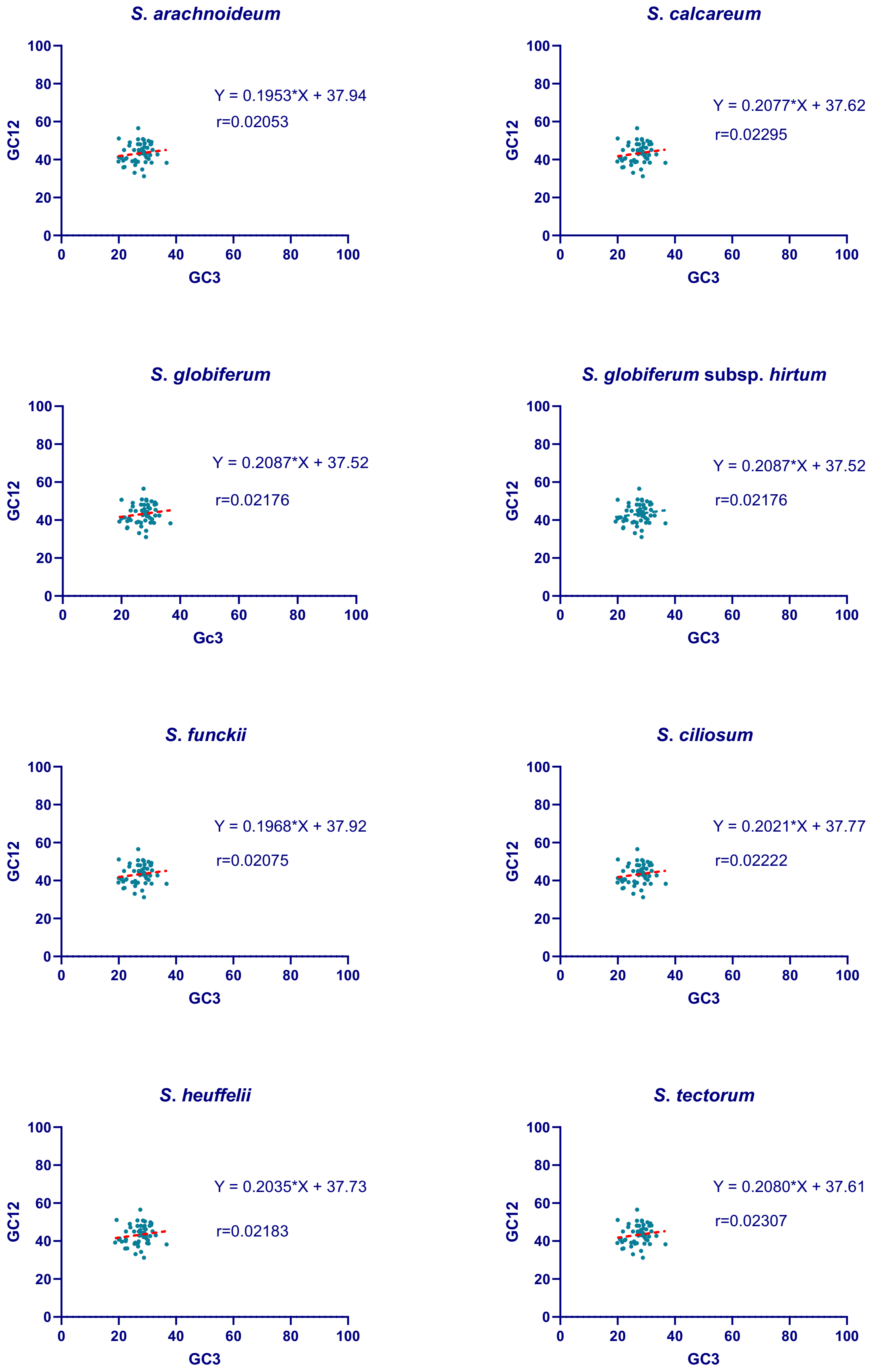
S. arachnoideum
Y = 0.1953*X + 37.94
r=0.02053
GC12
GC3
S. calcareum
Y = 0.2077*X + 37.62
r=0.02295
GC12
GC3
S. globiferum
Y = 0.2087*X + 37.52
r=0.02176
GC12
Gc3
S. globiferum subsp. hirtum
Y = 0.2087*X + 37.52
r=0.02176
GC12
GC3
S. funckii
Y = 0.1968*X + 37.92
r=0.02075
GC12
GC3
S. ciliosum
Y = 0.2021*X + 37.77
r=0.02222
GC12
GC3
S. heuffelii
Y = 0.2035*X + 37.73
r=0.02183
GC12
GC3
S. tectorum
Y = 0.2080*X + 37.61
r=0.02307
GC12
GC3
